# Analysis and Prediction of Influencing Factors of College Student Achievement Based on Machine Learning

**DOI:** 10.3389/fpsyg.2022.881859

**Published:** 2022-04-22

**Authors:** Dongxuan Wang, Dapeng Lian, Yazhou Xing, Shiying Dong, Xinyu Sun, Jia Yu

**Affiliations:** ^1^Department of Science and Technology, Hebei Agricultural University, Huanghua, China; ^2^College of Humanities and Management, Hebei Agricultural University, Huanghua, China; ^3^College of Mechanical and Electrical Engineering, Hebei Agricultural University, Baoding, China

**Keywords:** college student academic performance, factors analysis, the chi-square test, machine learning, analysis and prediction

## Abstract

To effectively improve students’ performance and help educators monitor students’ learning situations, many colleges are committed to establishing systems that explore the influencing factors and predict student academic performance. However, because different colleges have different situations, the previous research results may not be applicable to ordinary Chinese colleges. This paper has two main objectives: to analyze the fluctuation of Chinese ordinary college student academic performance and to establish systems to predict performance. First, according to previous research results and the current situation of Chinese college students, a questionnaire was designed to collect data. Second, the chi-square test was used to analyze the contents of the questionnaire and identify the main features. Third, taking the main features as input, four classification prediction models are established by machine learning. Some traits of the students who did not pass all the examinations were also discovered. It might help student counselors and educators to take targeted measures. The experiment shows that the support vector machine classifier (SVC) model has the best and most stable effect. The average recall rate, precision rate, and accuracy rate reached 82.83%, 86.18%, and 80.96%, respectively.

## Introduction

The development of a country depends on the cultivation of talent. Cultivating professional talent requires high-quality universities to improve student academic performance and strengthen student physical fitness to achieve general improvement of overall student quality. Therefore, how to enhance student learning has become a hot issue in the current education field. It is important to identify the influencing factors of college student academic performance to provide theoretical guidance for human intervention measures and students’ overall learning. In the past, the analysis of student academic performance and student learning methods was mainly based on teachers’ subjective judgment and decision making. This not only lacks attention to student ideas but also lacks scientific concepts and rigor, such that the overall result is unsatisfactory. In recent years, with the continuous expansion of enrollment in Chinese universities, the number of students has increased sharply. The scale of classroom teaching has also become increasingly large; it is difficult for teachers to track the learning progress of each student, which affects teaching quality. At the same time, a certain number of students in universities fail examinations, repeat grades, or even drop out of school, which seriously affects the development of higher education in China. In this context, it is of great applied value and practical significance to analyze the influencing factors of college student academic performance and to identify an efficient method for predicting student academic performance.

Student academic performance can be assessed by credit scores, rankings, or passing conditions, which all reflect a student’s learning status and level. For students, academic performance prediction plays a role in supervision and early warning. It helps students adjust learning methods and status in time, preventing failures as much as possible. Teachers can modify teaching strategies and teaching methods in a timely manner to increase the proportion of students who pass exams based on the actual situation. Counselors can pay more attention to students who are at risk of failing and the overall performance of students. For higher education institutions, it is possible to modify student training programs and methods to improve student learning outcomes.

In recent years, educational data mining has become one of the hottest topics in scholarly research. Data analysis is conducted with psychology and computer technology as fundamental by establishing predictive models based on machine learning knowledge for collecting student learning behavior data and data mining technology for extracting valuable educational information from data resources. It puts forward objective suggestions for student learning methods, teaching strategies, and educational models in universities; it even provides suggestions for improving overall student performance and making education more efficient. This study takes freshman students majoring in Computer Science in a Chinese university as a sample to carry out a questionnaire survey. The research subjects are divided into two categories—failing grades and passing grades—according to the influencing factors as the content of the questionnaire. The questionnaire’s content allows an analysis of the correlation between the influencing factors and the failure situation, the extraction of the main influencing factors, establishing a mathematical model with the failure behavior, and making targeted suggestions.

The contributions of this manuscript are as follows:

It compiles a questionnaire on metacognitive awareness, environmental factors, academic motivation, learning participation, and learning skills, which considers the literature on the overall history and the current situation in this university.

It classifies the influencing factors into primary, secondary, and irrelevant factors through the chi-square test. Then, 4 mathematical models are proposed that identify the primary factors and secondary factors as features and labels, respectively, to predict if the student can pass the exam successfully for early warning student learning effects.

It clarifies the evaluation criteria of the prediction model. The experimental results show that the prediction accuracy of SVC is the highest and the most stable.

Suggestions are put forward to students, teachers, and schools on multiple levels to improve college student academic performance and comprehensive quality according to the research conclusions.

The rest of the manuscript is organized as follows: in “Literature Review” section, related works are discussed, followed by an analysis of the influencing factors and the presentation of the mathematical model in “Factor Analysis and Mathematical Model Establishment” section. “Experimental Analysis” section discusses the experimental analysis and presents a comparative assessment, and the manuscript is concluded in “Conclusion” section.

## Literature Review

In recent years, research on student academic performance has been divided into studies on influencing factors and performance prediction.

Previous scholars have found that the factors that may affect student academic performance can be internal and external. Internal factors include learning motivation (Entwistle et al., 1971; Fortier et al., 1995), learning emotions (Shuyuan et al., 2012), learning behaviors (Jie, 2020), health status (Gilbert and Weaver, 2010; Wald et al., 2014; Wang et al., 2014; Faught et al., 2017), five major personality factors (Chamorro-Premuzic and Furnham, 2003; O’Connor and Paunonen, 2007; Poropat, 2009; Fu et al., 2012), and life behaviors (Hallinan, 2000; Wang et al., 2014; Almayan and Mayyan, 2016; Scanlon and Smeaton, 2017). External factors include family situation (parents’ educational level, occupation, etc.; Hallinan, 2000; Zhou, 2014; Almayan and Mayyan, 2016) and procrastination tendency (Hooshyar et al., 2019; Yang et al., 2020).

In 2018, Cao et al. (2018) extracted student behaviors from campus card data. Behaviors are divided into two types: study diligence and study order, including canteen eating, library access control, water fetching in the study room, and showering in the dormitory. Through the design of quantitative standards, it was found that there was, indeed, a degree of correlation between student behaviors and academic performance. In 2020, Jie (2020) integrated student behavioral data from the educational administration system, campus card, and campus Wi-Fi of 683 college students. He established quantitative standards and explored the relationship between student behavior and academic performance from study diligence and behavioral rules. These two studies show that academic achievement is related to behavioral factors, even though they lack subjectivity.

In 2018, Miguéis et al. (2018) conducted a study of 2,459 college students and divided student academic performance into five categories. He used data mining and machine learning algorithms to predict overall 4-year college academic performance using information available at the end of the first academic year. This paper has evident limitations: student academic performance fluctuates greatly, and there is uncertainty about future performance. In 2019, Quan et al. (2019) discovered that college student consumption was related to student academic performance, so they used the student’s campus consumption record and academic performance to establish a mathematical model to screen out students who may fail. In 2012, Huang and Fang (2013) collected 323 samples and developed 24 mathematical models to predict final-term dynamic grades by grade point average, four required courses, and three middle-term dynamic grades. The workload of this method is very large, but the achievement in a particular subject cannot represent overall learning performance, nor can it be used to predict overall performance. In the China Joint School Cooperation Program, the average learning level of students is relatively weak. Some students cannot graduate on time because their grades are not up to standard. In 2020, Hai-Tao et al. (2021) took the data of Shandong University of Science and Technology as an example and used the previous academic performance of students as input to set up an academic early warning system using convolutional neural networks. The methods have limitations, however, in that they can only be used in a small number of colleges.

Recently, researchers have tried to use data mining and machine learning techniques to predict student academic performance (Hooshyar et al., 2019; Jie, 2020; Yang et al., 2020). Taking 2,039 students from 2016 to 2019 as a sample, it predicts the grades after admission through the grades before admission. It provides a theoretical basis for the admission process of universities (Mengash, 2020). This study shows that pre-university performance can be used to predict performance once a student is enrolled in university. However, the effect may not be satisfactory. Jun Li and others collected 473 questionnaires from Wuhan university students to investigate the relationship between self-directed learning, optimism, mental health, and academic performance. Research shows that academic achievement can be directly influenced by self-directed learning and indirectly influenced by optimism and mental health. However, the defect of this study is that there are few influencing factors to predict academic achievement; furthermore, it only applies to specific college students (Li et al., 2021). Mohamed Jaber et al. proved that students’ personality profiles are an important predictor of academic achievement by using the Big Five Inventory (BFI) scale. However, the subjects of the study were limited to medical college students over 24 years old, which is not applicable to all college students (Jaber et al., 2022).

Academic performance is indeed related to learning motivation, learning emotions, learning behaviors, health status, five major personality factors, and life behavior. However, there are still problems to be solved. On the one hand, predicting the final grade in a certain subject does not reflect student learning status, which has certain limitations. On the other hand, the factors influencing student performance differ in different countries’ universities and in universities of different levels, so the existing research findings are not fully generalizable to ordinary Chinese universities. Therefore, determining the main influencing factors of Chinese college students’ academic performance and establishing an appropriate performance early warning system is the main research purpose in this paper.

## Factor Analysis and Mathematical Model Establishment

### Chi-Square Test

One of the purposes of this paper is to establish the correlation between questionnaire questions and failing exams. Analyzing the degree of correlation among variables mainly includes the Pearson coefficient method, the Kendall rank correlation coefficient method, and the Spearman correlation coefficient method. Since the variables in the questionnaire of this study are discrete and nonequidistant categorical, the Pearson chi-square test was selected for the preselection of factors.

In October 2020, Zander et al. (2020) used the chi-square test to show that effective mastery and cognitive self-enhancement are correlated in predicting self-efficacy in girls and boys. In 2019, Danxing (2019), from Jinan University, used the chi-square test to analyze the correlation between their classroom accumulation, intensive review before the final and college students’ English final scores. In this paper, the Pearson chi-square test is used to determine the relationship between each feature in the questionnaire and failure, that is, whether the variables of the questions in the questionnaire are related to the variable of failure determines the inputs in the prediction model. The detailed steps are as follows:

Step 1: *Hypothesis H0*: the distribution of the two variables is inconsistent.

Step 2: Calculate the cross-table of the two variables (as shown in Table 1) and the expected frequency (as shown in Formula 1).


(1) $$T_{ij}=\frac{\left(\sum_{j=0}^{1}x_{ij}\right)\left(\sum_{i=A}^{E}x_{ij}\right)}{\sum_{i=A}^{E}\sum_{j=0}^{1}x_{ij}}$$

**Table 1:** Cross-tab.

	0	1	Total
A	$x_{A0}$	$x_{A1}$	$x_{A0}+x_{A1}$
B	$x_{B0}$	$x_{B1}$	$x_{B0}+x_{B1}$
C	$x_{C0}$	$x_{C1}$	$x_{C0}+x_{C1}$
D	$x_{D0}$	$x_{D1}$	$x_{D0}+x_{D1}$
E	$x_{E0}$	$x_{E1}$	$x_{E0}+x_{E1}$
Total	$x_{A0}+\cdots +x_{E0}$	$x_{A1}+\cdots +x_{E1}$	$x_{A0}+\cdots +x_{E0}+x_{A1}+\cdots +x_{E1}$

*x_ij_* *indicates the frequency of students choosing option *i* under the condition of *j*. Among them, *i* = A, B, C, D, E represents the options of the questionnaire; *j* = 0,1 represents all students pass and not all students pass the final exam*.

In Formula 1, T*_ij_* represents the expected frequency.

Step 3: If n (the number of samples) >40 and T*_ij_* > 5, the next step can be continued; otherwise, a continuous corrected chi-square test or Fisher test is needed. (These data meet the above conditions, so the continuous correction chi-square test and Fisher test will not be introduced.)

Step 4: Calculate the value of *p* and statistical value according to the cross-tab. The value of *p* represents the probability that the distributions of the two variables are inconsistent. The calculation method of $\chi^{2}$ is shown in Formula 2.


(2) $$\chi^{2}=\overset{E}{\underset{i=A}{\sum }}\overset{1}{\underset{j=0}{\sum }}{\frac{\left(x_{ij}-T_{ij}\right)}{T_{ij}}}^{2}$$

Step 5: Check the critical value table to observe whether the chi-square test is statistically significant. That is, the distribution of the two variables is consistent. The chi-square statistic value is large enough (or the value of *p* is small enough), and the correlation between the two variables is strong.

### Mathematical Model

At present, popular machine learning classification techniques mainly include the logistic regression (LOG) model, support vector machine classifier (SVC) model, random forest classifier (RFC) model, and naive Bayes classifier (NBC) model.

The LOG model (Yuan, 2021) is a supervised machine learning algorithm generally used for dichotomous prediction. The value of the independent variable is a random real number, and the output is [0,1]. The core idea is to calculate a predicted value in linear regression and map the predicted value to the sigmoid function to complete the conversion from the value to probability. By default, the threshold is 0.5. If P is greater than 0 but less than or equal to 0.5, it is classified as 0. If P is greater than 0.5 but less than or equal to 1, it is classified as 1. The threshold can be changed according to the actual situation, which changes the classification result. The algorithm is simple and fast, but the prediction effect is sometimes not good enough.

The SVC model (Yang et al., 2020) is a supervised machine learning algorithm used for classification prediction. The core idea of the SVC classifier is to find an optimal hyperplane in the high-latitude space to divide the data to maximize the margin. The algorithm is simple, fast, and suitable for a small sample. However, the predicted results are only 0 and 1, which are different from the LOG model in that the predicted probability threshold can be modified.

The RFC model (Wu et al., 2022) is an integrated learning method based on a decision tree in machine learning. It is generally used for classification prediction more than regression prediction. In classification prediction, each tree is a classifier, each classifier has a classification result, and the class with the most classification results is set as the final output. In the face of high dimensionality, there is no need to reduce dimensionality, and the computation between trees is parallel. However, in the case of a small sample or low latitude, performance is not good enough to produce a good classification effect.

The NBC model (Chen et al., 2021) is a Bayesian classifier, which is a machine learning classification algorithm in statistics and probability knowledge. According to the Bayesian formula, the prior probability is calculated, and then, the posterior probability is obtained. If the posterior probability belongs to a class with a high probability, it shall be classified as this class. The method is simple, the classification accuracy is high, and the calculation speed is fast, but there are limitations to assuming that the attributes are independent, which is inevitable in practical applications so that the classification effect may be affected.

### Model Evaluation Criteria

This study belongs to a dichotomous problem. The commonly used evaluation criteria for classification models are based on the confusion matrix (as shown in Table 2), mainly composed of accuracy, precision, recall, and the ROC curve.

**Table 2:** Confusion matrix.

True value	Predict value	
	P′	N′
P	TP	FN
N	FP	TN

Accuracy is the percentage of the total number of correct predictions.

TP: the number of samples for which both the predicted and true values are 1; TN: the number of samples for which both the predicted and true values are 0; FP: the number of samples for which the true value is 0 and the predicted value is 1; and FN: the number of samples for which the true value is 1 and the predicted value is 0.


(3) $$\mathrm{accuracy}=\frac{TP+TN}{TP+TN+FN+FP}$$

Precision refers to the proportion of the number of samples in which the predicted and true values are both 1 to the number of samples in which the predicted value is 1.


(4) $$\mathrm{precision}=\frac{TP}{TP+FP}$$

In this study, students with failed records account for a small proportion, so the accuracy rate and precision rate do not reflect the effect of the model well; thus, the recall rate needs to be introduced. The recall rate refers to the ratio of the number of samples with predicted and true values of 1 to the number of samples with true values of one.


(5) $$\mathrm{Recall}=\frac{TP}{TP+FN}$$

The ROC curve takes the true positive probability as the ordinate and the false-positive probability as the abscissa. In grade prediction, the ordinate represents the probability that a student will fail, while the abscissa represents the probability of a student failing being wrongly predicted. The closer the curve is to the vertical coordinate, the higher the prediction accuracy is, and the better the model effect is.

## Experimental Analysis

### Data Sources

According to previous research results and the actual situation of students in ordinary Chinese universities, the contents of the designed questionnaire mainly include metacognitive awareness, environmental factors, learning motivation, learning participation, learning skills, and methods, as shown in Tables 3–6. The questionnaire contains 27 multiple-choice questions.

**Table 3:** Questions about metacognitive awareness.

Number	Question
Q1	Are you satisfied with your college entrance examination results?
Q2	How do you feel about your sense of achievement during your freshman year of study?
Q3	Do you think you received enough attention from your teachers during freshman year?
Q4	How often did you feel depressed during your freshman year?
Q5	How do you feel about the stress of your freshman year?
Q6	How much did you absorb what your teacher taught you in class during your freshman year?

**Table 4:** Questions about environmental factors.

Number	Question
Q7	Where does your family (growth environment) come from?
Q8	What is your parents’ highest educational degree?
Q9	What do you think of the learning atmosphere in your dormitory?
Q10	How do you feel about your relationship with your classmates and roommates?

**Table 5:** Questions about learning motivation.

Number	Question
Q11	How do you feel about your interest in computer science?
Q12	What do you do when you have a problem with your studies during your freshman year?
Q13	How useful do you think textbook knowledge will be in your future work?
Q14	What do you think is your motivation to study?
Q15	What are your plans for your future?

**Table 6:** Questions about learning participation skills.

Number	Question
Q16	What do you think of your schedule?
Q17	How often do you preview before class?
Q18	How often do you review after class?
Q19	How much effective self-study time did you spend on average every day during your freshman year?
Q20	How often do you ask your teacher questions after class?
Q21	How many times did you participate in entrepreneurship and innovation competitions during your freshman year?
Q22	How often did you skip classes or find an excuse to skip class during your freshman year?
Q23	Where did you sit most of the time in the class during your freshman year?
Q24	How long can you concentrate in class? (total 45 min)
Q25	How often did you sleep in class during your freshman year?
Q26	How often did you play with your mobile phone in class during your freshman year?
Q27	How did you finish the homework assigned by the teacher during your freshman year?

After collecting the questionnaire, we found that some of the contents were invalid. Therefore, we eliminated this group of students from the sample. Finally, we acquired 292 valid samples. The grades of 292 freshmen in each subject are derived through the educational administration system. We use 0 to express all passes and 1 to express all fails. Some of the datasets are shown in Table 7.

**Table 7:** Partial dataset.

Name	Q1	Q2	Q3	Q4	Q5	……	Q23	Q24	Q25	Q26	Q27	Pass_or_fail
Zhang Jiankun	B	A	A	B	C	…	B	B	A	B	C	1
Zhang Jinxing	B	B	B	C	B	…	B	B	B	B	B	0
Ma Xiao	C	B	B	C	A	…	B	B	B	A	A	0
Su Meiqi	A	B	B	B	C	…	C	C	B	C	B	1
Fu Haobin	C	B	B	B	A	…	B	B	B	B	B	1

**Figure 1:**
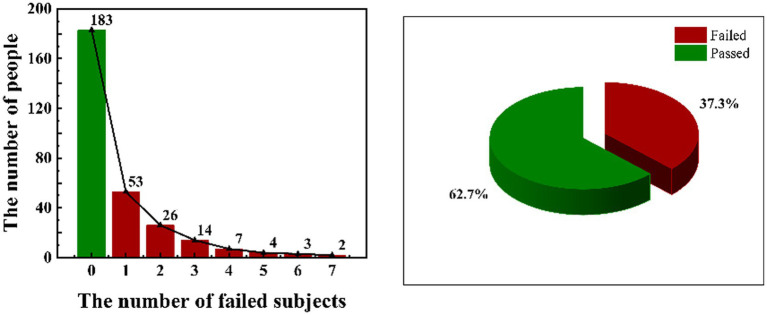
An overall academic performance.

To grasp the academic situation of the research subjects, we organized and analyzed their overall academic performance. From Figure 1, we found that 183 students passed all final exams and 109 students failed at least 1 subject, accounting for 62.2% and 37.8% of the total, respectively. Among students with failed subjects, 54 students failed 1 subject, 26 students failed 2 subjects, 21 students failed 3–4 subjects, 7 students failed 5–6 subjects, and only 2 failed 7 subjects. Overall, more than one-third of the students failed records. The number of failed subjects is in inverse proportion to the number of students.

### Chi-Square Test Results

Through the chi-square test, we accurately screen out the features having a strong correlation with failure as independent variables and the failure record as the dependent variable. The results of the cross-tab and expected frequency calculation of Q1–Q5 are shown in Tables 8 and 9, where T is greater than 5, and the sample number of this study, *n* = 292, is greater than 40, so the Pearson chi-square test can be adopted. Table 10 is the chi-square test results between 27 questions and failing records, where the value of *p* is less than 0.01 between Q1, Q2, Q6, Q11–Q13, Q15, Q18, Q20, Q22–Q24, and Q27 and failing records, indicating a strong correlation and they are the primary influencing factors. The correlation between Q4, Q5, Q16, Q19, and Q26 and failure record is 0.01 < *p* < 0.05, which means that the correlation is general and they are secondary influencing elements. For the remaining features, *p* > 0.05 means they are not correlated with the failure records. Due to the large data dimension, only the features Q1–Q5 are given in Tables 8 and 9, and the remaining tables are no longer listed.

**Table 8:** Cross-tab statistics of the Q1–Q5 questionnaires.

Question	Q1		Q2		Q3		Q4		Q5	
Option	0	1	0	1	0	1	0	1	0	1
A	7	16	63	14	34	22	16	11	12	12
B	40	40	92	58	149	88	149	79	84	33
C	61	39	19	22	–	–	18	20	78	58
D	61	9	9	16	–	–	–	–	9	7
E	14	6	–	–	–	–	–	–	–	–

**Table 9:** Expected frequency T.

Question	Q1		Q2		Q3		Q4		Q5	
Option	0	1	0	1	0	1	0	1	0	1
A	14.37	8.63	48.09	28.91	34.98	21.02	16.86	10.14	14.99	9.01
B	49.97	30.03	93.69	56.31	148.02	88.98	142.40	85.60	73.08	43.92
C	62.46	37.54	25.61	15.39	–	–	23.73	14.27	84.94	51.06
D	43.72	26.28	15.61	9.39	–	–	–	–	9.99	6.01
E	12.49	7.51	–	–	–	–	–	–	–	–

**Table 10:** Chi-square test results (per the chi-square test critical value).

Q	*χ* ^2^	*p*	*R*	*df*	0.05 Critical value	Q	*χ* ^2^	*p*	*R*	*df*	0.05 Critical value
1	34.12	7.04E-07	**	4	9.488	15	85.62	1.90E-18	**	3	7.815
2	24.39	2.07E-05	**	3	7.815	16	6.83	0.07751	*	3	7.815
3	0.02	0.883851	–	1	3.841	17	0.96	0.32532	–	1	3.841
4	4.62	0.099183	–	2	5.991	18	15.52	0.000425	**	2	5.991
5	7.71	0.052331	–	3	7.815	19	7.83	0.049628	*	3	7.815
6	44.51	2.16E-10	**	2	5.991	20	19.25	1.15E-05	**	1	3.841
7	0.98	0.612043	–	2	5.991	21	0.20	0.654369	–	1	3.841
8	2.18	0.335724	–	2	5.991	22	63.19	1.89E-14	**	2	5.991
9	4.41	0.10994	–	2	5.991	23	55.51	8.81E-13	**	2	5.991
10	0.81	0.665165	–	2	5.991	24	32.67	3.78E-07	**	3	7.815
11	45.34	7.80E-10	**	3	7.815	25	4.39	0.110819	–	2	5.991
12	9.26	0.002339	**	1	3.841	26	7.87	0.019511	*	2	5.991
13	16.96	0.000717	**	3	7.815	27	58.20	2.29E-13	**	2	5.991
14	4.91	0.177763	–	3	7.815						

*R* represents the degree of correlation, and *df* represents the degree of freedom. *p* > 0.05 indicates no correlation between variables represented by –; 0.1 < *p* < 0.05 indicates a moderate correlation between the two variables represented by *; and 0 < *p* < 0.01 indicates a strong correlation between the two variables represented by **.

The chi-square test results show that the influencing factors of student academic performance in our school mainly came from metacognitive awareness, learning motivation, learning participation, and learning skills. Metacognitive awareness includes college entrance examination score satisfaction and self-achievement. Learning motivation includes learning interest and future work plans. Learning participation and learning skills include the frequency of reviewing material, the frequency of consulting the teacher, the quality of completed homework, the concentration in class, and the seat in the classroom.

### Model Prediction Results

When modeling, the label indicates whether the final exam is failed, with a value of 1 or 0. The features are the questionnaire questions, the primary influencing factors affecting student academic performance that pass the chi-square test. Since the question options in the questionnaire are not equidistant and cannot be converted into numerical values, one-hot encoding is used to solve this problem. One-hot encoding is a representation that regards categorical variables as binary vectors. For example, there are five options, (A, B, C, D, E), in Question 1 of the questionnaire. According to the one-hot coding rule, each option is coded as [(0,0,0,0,1), (0,0,0,1,0), (0,0,1,0,0), (0,1,0,0,0), or (1,0,0,0,0)].

Usually, before model training, the original data are divided into the training set and the testing set. The original data cannot be divided because of the minute dataset that has failed subjects, which may lead to model overfitting or underfitting. In the traditional method of separating data, oversampling and undersampling are usually used to increase the number of samples to balance the number of positive and negative samples, but there are limitations about nonideal effects.

**Figure 2:**
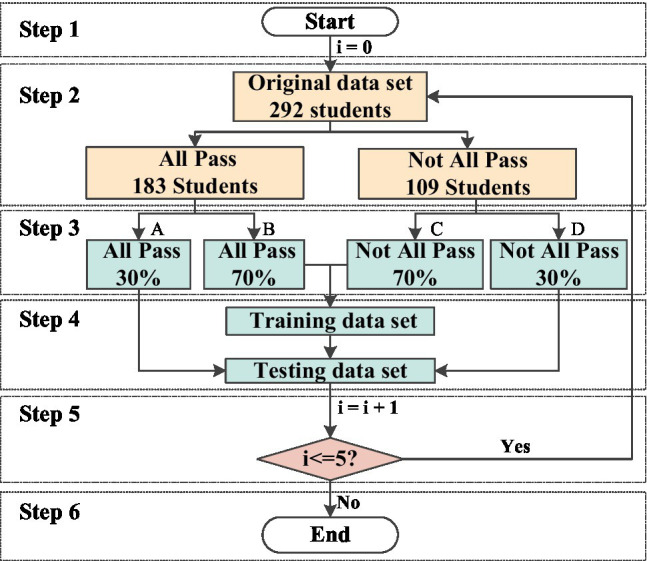
The flow chart of dataset segmentation.

When separating the training and testing sets of the model, the idea of stratified sampling is introduced. As shown in Figure 2, the concrete flow chart is as:

Step 1: *i* refers to the number of loops; *i* = 0.

Step 2: The original dataset is divided into an all pass set (183 students) and a not all pass set (109 students).

Step 3: All pass sets are randomly divided into A sets with 30% data and B sets with 70% data. Not all pass sets are randomly divided into C sets with 70% data and D sets with 30% data.

Step 4: The training dataset is a combination of sets B and C. The testing dataset is a combination of set A and set D.

Step 5: If *I* < =5, return to step 2; otherwise, go to the next step.

Step 6: End.

On the premise that the training set is the same as the test set, the representative LOG, SVC, RFC, and BBN in the binary classification model are used. The machine learning model is implemented using “sklearn” in Python. Make use of the “sklearn linear_ model; Logistic ()” to create LOG model object; make use of “sklearn.svm.svc()” to create the SVC model object; make use of “sklearn.ensemble; RandomForestClassifier()” to create the RFC model object; and make use of “sklearn.naive_bayes.BernoulliNB()” to create the BNB model object. Then, the separated training set and test set are used to train the model and verify the model.

In the SVC model, the parameters C, kernel, and gamma usually need to be adjusted. The larger C is the larger the punishment for the wrong sample; therefore, the greater the accuracy in the training set. However, the generalization ability is reduced, versus the classification accuracy of the test data being reduced. In contrast, if C is reduced, some misclassified samples are allowed in the training set. This may lead to enhanced generalization. Therefore, to prevent overfitting and underfitting, C should not be too large or too small. The kernel is used to select different kernel functions as decision boundaries. It can be set as one of “linear,” “poly,” “rbf,” “sigmoid,” and “precomputed.” The parameter gamma mainly maps the low dimension to the high dimension. The higher the gamma value is, the higher the mapping dimension, and the better the training result. A large gamma will lead to a higher dimension of mapping and a better training result. However, it is easy to cause overfitting; that is, the generalization ability is low. Under the condition of simultaneously ensuring the accuracy of the training set and test set, the grid search method was used to obtain the best parameters of *C* = 1.0 and kernel = “linear.” Because the kernel is “linear,” gamma does not need to be set.

Accidentally, the model may perform well in some specific training sets and test sets. Therefore, to eliminate this contingency and ensure feasibility, stability, and reproducibility, we conducted six random experiments.

**Figure 3:**
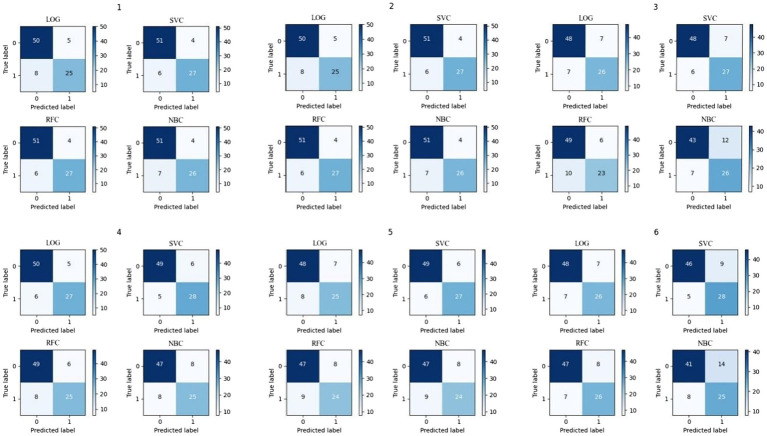
The confusion matrix.

**Figure 4:**
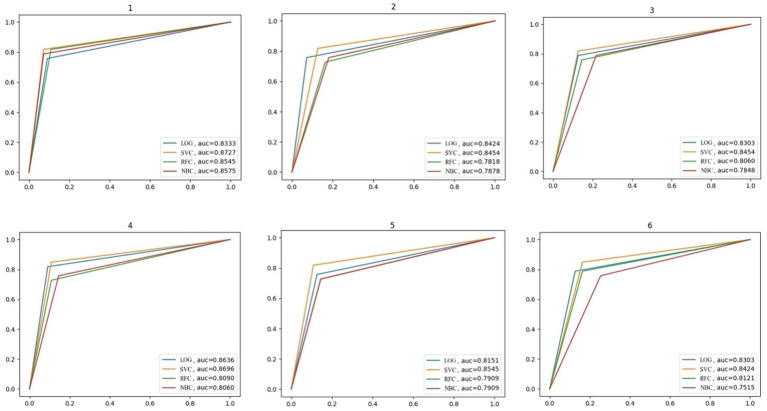
The ROC curve.

The confusion matrix is shown in Figure 3, and the recall, accuracy, and precision values are shown in Table 11. The ROC curve, based on the confusion matrix, is drawn in Figure 4 to evaluate the effectiveness of the models in predicting students’ academic performance.

**Table 11:** Model prediction results.

Evaluation criterion	Models	1 (%)	2 (%)	3 (%)	4 (%)	5 (%)	6 (%)	Average (%)	*R* ^2^
Recall	LOG	75.76	75.76	78.79	81.82	75.76	78.79	77.78	0.0247
	SVC	81.82	81.82	81.82	84.85	81.82	84.85	82.83	0.0156
	RFC	81.82	69.70	75.76	75.76	72.73	78.79	75.76	0.0429
	BNB	78.79	75.76	78.79	75.76	72.73	75.76	76.27	0.0228
Accuracy	LOG	85.23	86.36	84.09	87.50	82.95	84.09	85.04	0.0167
	SVC	88.64	85.23	85.23	87.50	86.36	84.09	86.18	0.0167
	RFC	88.64	81.82	82.95	85.23	80.68	82.95	83.71	0.0285
	BNB	87.50	79.55	78.41	81.82	80.68	75.00	80.49	0.0415
Precision	LOG	83.33	86.21	78.79	84.38	78.13	78.79	81.61	0.0346
	SVC	87.10	79.41	79.41	82.35	81.82	75.68	80.96	0.0382
	RFC	86.67	73.33	76.67	82.35	75.00	76.47	78.42	0.0506
	BNB	86.67	71.43	68.43	75.76	75.00	64.10	73.57	0.0773

As seen from the average column in Table 11, the average recall and accuracy of the SVC model are the highest, reaching 82.83% and 86.18%, respectively. The average precision of the LOG model is the highest, reaching 81.61%. Moreover, the recall, accuracy, and precision of the SVC model are the minimum. Meanwhile, Figure 4 illustrates that the SVC model has the highest AUC of the ROC curve. In summary, the SVC model shows the best classification effect on this dataset.

Compared with other classifiers, SVC is a more suitable dichotomous classification. It provides overfitting, noise data, and outlier processing. In addition, it also has the advantages of being fast, effective in high-dimensional spaces, and the ability to provide different kernel functions as decision functions.

The SVC classification algorithm proposed in this paper is suitable for ordinary Chinese universities, such as Hebei Agricultural University, to establish an academic early warning system. Because the research object of this study is 292 freshmen from Hebei Agricultural University, the SVC classification algorithm proposed in this paper is suitable for ordinary Chinese university equivalents similar to Hebei Agricultural University, to establish an academic early warning system. It may not be applicable to China’s double first-class universities or to foreign universities. The research results have some limitations, but the research method is universal. According to this method, we can study the influencing factors of student academic performance in different universities and establish an academic early warning system.

## Conclusion

Students’ satisfaction with college entrance examination results, self-achievement, and knowledge absorption in class have an impact on freshman academic performance. We speculate that college entrance examination results have a self-directed impact on students. The sense of self-achievement and knowledge absorption in class have a psychological impact on students. Therefore, we believe that students’ satisfaction with college entrance examination results implies students’ self-examination in the previous stage. Students’ self-achievement and knowledge absorption in class reflect students’ self-evaluation in the learning stage. As the coverage point of metacognitive consciousness, a combination of all these factors has a certain impact on students’ academic performance.

Learning motivation has a great impact on student academic performance. In psychology, demand is the source of all behaviors. It becomes the internal driving force to promote and maintain individual behavior when the demand translates into motivation. In addition, we believe that the learning goal, the appreciation of academic content, and the ability to plan the learning process also provide students with indispensable learning motivation. These findings are confirmed in our results.

It has also been proven that learning skills are one of the effective factors affecting college student academic performance. In terms of academic participation, this paper analyzes the students’ failure from the perspectives of preview and review, asking questions and skipping classes. The results show that the greater student academic participation, the lower the probability of failure.

In addition, we find that students’ class state, including attention and time concentrating in class and homework completion after class, has different degrees of impact on students’ failure. In particular, we found that there was a strong correlation between students’ seating position in class and failure when the seats were divided into front, middle, and rear. This can be explained by the fact that where students sit in the classroom can represent their learning enthusiasm; thus, it has a certain impact on academic performance.

However, the study found some inconsistencies with previous research conclusions. For example, depression does not necessarily lead to failure because most anxiety comes from learning pressure, which can be effectively transformed into learning motivation. In addition, academic performance of the study object has no direct relationship with the surrounding environment. These findings all provide a novel direction for future research.

Moreover, there are some specific suggestions for students, teachers, and institutions based on the conclusions. Students should set academic goals and make plans. They should be humble in victory and gracious in defeat. When faced with difficulties in the learning process, they can try to solve them themselves or actively ask teachers for help. Teachers should appraise or criticize students appropriately to enhance students’ sense of self-achievement. They should be devoted to strengthening communication with students, cultivating their professional interests, and guiding them to study independently. Colleges and universities should stimulate students’ learning motivation, cultivate students’ independent scientific research ability, and create a positive and orderly university learning atmosphere to actively respond to national policies.

## Data Availability Statement

The original contributions presented in the study are included in the article/supplementary material; further inquiries can be directed to the corresponding author.

## Author Contributions

DW and DL: writing. YX and SD: translating. XS and JY: editing. All authors contributed to the article and approved the submitted version.

## Funding

This work was supported in part by the Bohai College of Hebei Agricultural University (grant no. 2021-BHXT-17), and by the Research Foundation of Education Bureau of Hebei Province of China (grant no. QN2019184).

## Conflict of Interest

The authors declare that the research was conducted in the absence of any commercial or financial relationships that could be construed as a potential conflict of interest.

## Publisher’s Note

All claims expressed in this article are solely those of the authors and do not necessarily represent those of their affiliated organizations, or those of the publisher, the editors and the reviewers. Any product that may be evaluated in this article, or claim that may be made by its manufacturer, is not guaranteed or endorsed by the publisher.
